# A review of influenza detection and prediction through social networking sites

**DOI:** 10.1186/s12976-017-0074-5

**Published:** 2018-02-01

**Authors:** Ali Alessa, Miad Faezipour

**Affiliations:** 1 Department of Computer Science and Engineering, School of Engineering, University of Bridgeport, 221 University Avenue, Bridgeport, 06604 CT USA; 2 Department of Biomedical Engineering, School of Engineering, University of Bridgeport, 221 University Avenue, Bridgeport, 06604 CT USA

**Keywords:** Flu trend, Social media data, Illness Like Influenza (ILI)

## Abstract

Early prediction of seasonal epidemics such as influenza may reduce their impact in daily lives. Nowadays, the web can be used for surveillance of diseases. Search engines and social networking sites can be used to track trends of different diseases seven to ten days faster than government agencies such as Center of Disease Control and Prevention (CDC). CDC uses the Illness-Like Influenza Surveillance Network (ILINet), which is a program used to monitor Influenza-Like Illness (ILI) sent by thousands of health care providers in order to detect influenza outbreaks. It is a reliable tool, however, it is slow and expensive. For that reason, many studies aim to develop methods that do real time analysis to track ILI using social networking sites. Social media data such as Twitter can be used to predict the spread of flu in the population and can help in getting early warnings. Today, social networking sites (SNS) are used widely by many people to share thoughts and even health status. Therefore, SNS provides an efficient resource for disease surveillance and a good way to communicate to prevent disease outbreaks. The goal of this study is to review existing alternative solutions that track flu outbreak in real time using social networking sites and web blogs. Many studies have shown that social networking sites can be used to conduct real time analysis for better predictions.

## Background

Public health is an important issue. Health care providers should be updated about the public health and disease outbreaks affecting their communities in order to make correct decisions at the right time. This would help them offer better services in an efficient way and at the perfect time. Most of the health care providers depend on the Center of Disease Control and Prevention (CDC) to be informed about disease outbreaks or to be notified about the flu season.

The Center of Disease Control and Prevention (CDC) is a trusted department in the United States. It publishes weekly disease related reports. One of the weekly reports is the influenza epidemic report. The CDC publishes flu-related reports using United States Influenza Like Illness Surveillance Network (ILINet) that gathers flu-related information of outpatients from hundreds of healthcare providers around the states. ILINet shows accurate results in detecting flu outbreaks, but it is costly and takes a long time to issue the required reports. Details of collecting and using CDC ILINet is discussed in [1].

Since we live in the data era, social networking sites (SNS) are widely used to post news, events, and even to express feelings. Therefore, SNSs have played an important role in real time analysis and have been used for faster trend predictions in many areas [2, 3]. The areas include traffic [4–7], disaster prediction [8–12], management [13–15], networking [16, 17], news [18–22] and so on. In the public health area, SNS provides an efficient resource for disease surveillance and also an efficient way to communicate to prevent disease outbreaks [23]. Early detection of seasonal epidemics such as influenza may reduce its impact. The use of SNS data to detect the spread of epidemics such as flu in the population, can help to obtain early warnings. SNS users can be used as sensors that provide data to be analyzed for early trend detections and predictions. New techniques for analysis on search engine logs [24–29] and social media data can be used to get real time analysis for better services [30].

Based on our survey of disease outbreak detection models using social media data, we found that most studies and models were developed to detect influenza outbreak from SNS such as seasonal influenza and the swine influenza. The developed models can potentially be deployed for other disease outbreak detections and predictions. Although prediction and detection terms are used throughout the review, the terms have different definitions. Flu detection refers to the process of discovering flu cases that already occurred. On the other hand, flu prediction collects data to predict flu trends. Furthermore, the term nowcasting refers to the process of predicting flu cases that happened in real time, which surveillance systems overlook. Due to the surveillance system limitations, the need for new techniques and models, such as Google Flu Trend (GFT) are necessary in order to predict non-reflected flu cases. This nowcasting process is integrated into report revisions before the final reports are issued. Aside from nowcasting, the process of forecasting is used to predict real flu cases in the future.

Most studies use the Twitter micro blog because it is the most widely used social networking site. It is an efficient resource to track trends for several reasons. First, the high frequency of posted messages helps to perform minute-by-minute analysis. Second, compared with search engine logs, Twitter posts are more descriptive and available for the public. In addition, more analysis can be performed by analyzing the users’ profiles such as demographic data and specific details. Third, users of Twitter are of diverse ages, not only young people, but also middle aged, and technology savvy older population [31].

The focus of this paper is to survey the existing tools, techniques, frameworks, and methods of predicting influenza trends in social media data. The studied methods evaluate the Twitter posts that have keywords related to influenza for faster detection in an effort to achieve and maintain healthier communities.

The rest of the paper is organized as follows. The “Article selection methodology and related work” section first presents the method of article selection and evaluation for this review in addition to the related work. The “Methods” section, then, comprehensively demonstrates different methodologies and techniques of influenza trends detection from social media data. The “Discussion” section presents a discussion and comparison among all the proposed existing methodologies. Then, the “Challenges” section discusses the challenges of using social media data for detection processes. Finally, concluding remarks and future directions appear in the “Conclusion” section.

## Article selection methodology and related work

This review paper aims to review the published work in the past recent years that use social media data such as Twitter to detect influenza. Relevant articles were collected from various resources and publishers including IEEE, ACM, BMC, and MDPI. Different keywords were used to collect the relevant articles such as “Influenza trend prediction using social media data”. During the collection process the initial number of retrieved articles was 671. The selection process was based on certain criteria such as:

- Being relevant to flu outbreak detection and prediction
- Analyzing social media data in the detection and prediction process
- Being in English Language.

**Fig. 1:**
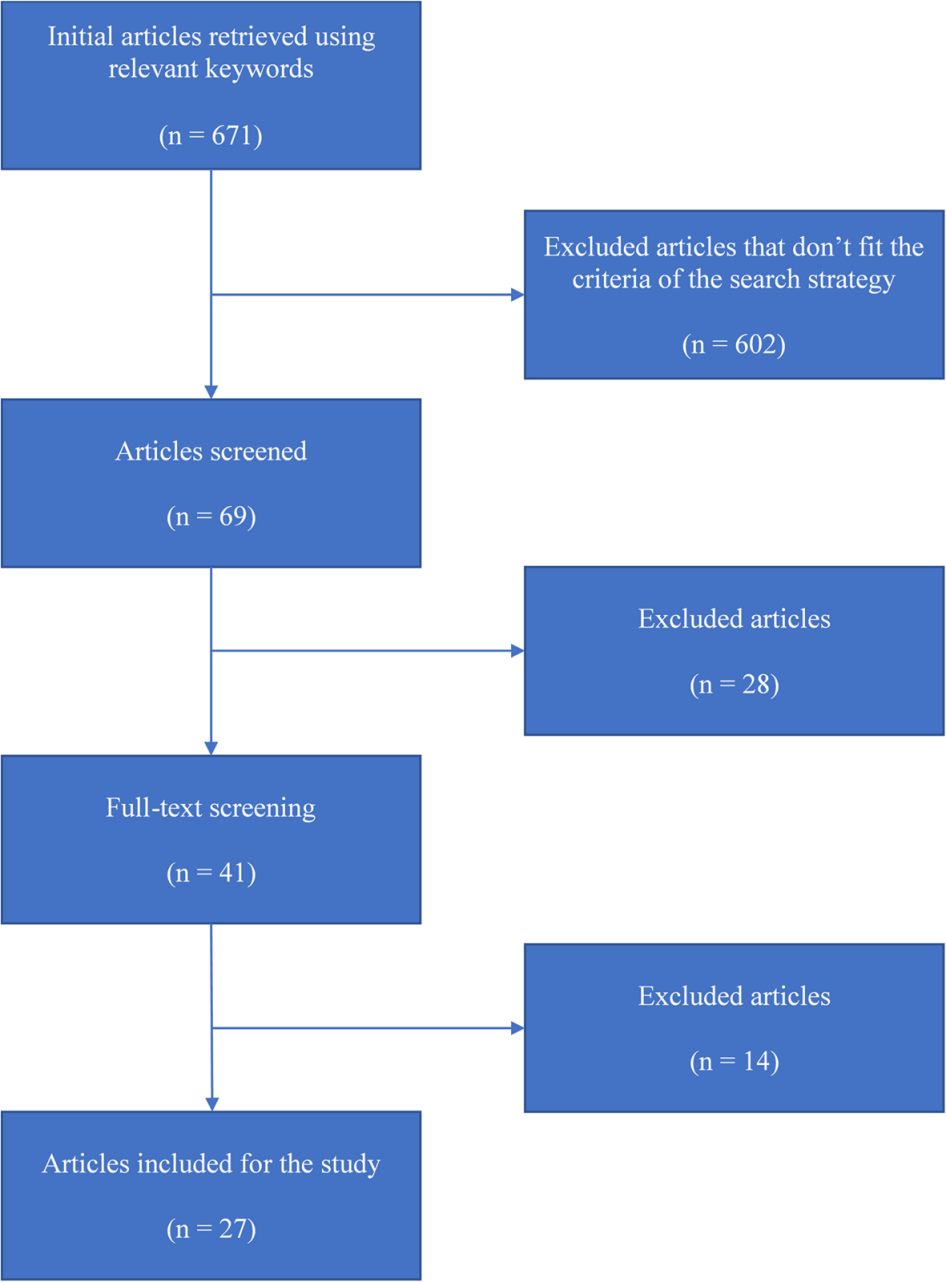
Articles selection process. The figure shows the general overview of the used methodology of article selection

Based on the selection criteria, 602 articles were excluded by reviewing the titles and the abstract of the retrieved articles. Initially, the selected articles were reviewed entirely. Out of 69 of the selected articles, 41 articles satisfied all the criteria. The final number of selected articles that were considered for this review was 27 articles. The other 14 articles were insufficient. Figure 1 summarizes the process of article selection.

Several prediction and detection models that are using other web data, such as Google Flu Trend (GFT), have been published in the literature for flu outbreak prediction and detection. Some of these models, such as PROFET, are included in this review to clarify that they can potentially work with the available social media data. Some other publications in the literature present flu surveillance related tools and web applications that don’t use social media data for flu detections and predictions. Some of these applications and tools are listed below:

- FluNearYou (https://flunearyou.org/): FluNearYou [32] is a web application that uses weekly surveys to collect health status of individuals in addition to the data obtained from CDC and GFT. By using the data from the three sources, the application shows the spread of the disease in the form of maps and charts.
- Influenzanet (http://www.influenzanet.eu): Influenzanet [33] is a web application that collects real-time data about flu epidemics in several European countries through more than 30,000 contributors of internet volunteers. Volunteers are asked to report their status weekly.
- FluOutlook (https://fluoutlook.org/): FluOutlook [34] is a web application that shows forecasts of the current flu season in North America and Europe in form of maps and charts. Reports are updated weekly using CDC reports. FluOutlook is based on the compartmental epidemic model.
- Columbia Prediction of Infectious Diseases (http://cpid.iri.columbia.edu/): Columbia Prediction of Infectious Diseases is a web application that shows forecasts of seasonal flu in curve charts. It also shows the current ILI counts in the US in a map format [35].
- HealthMap (https://www.healthmap.org/): HealthMap is an infectious disease monitoring system. It uses unstructured reports of the infectious diseases from multiple sources in the internet, filters them, classfies and visualizes information about important identified disease outbreaks [36].

## Methods

There are many ways to discover knowledge and predict flu trends from Twitter data. This section glances at various existing techniques. The studies for this review were selected to include the existing methods and techniques applied to SNS data for earlier influenza outbreak prediction. The studied methods and techniques are within the past recent years that fall under one of the main categories of graph data mining, text mining, topic models, machine learning, math/statistical models or mechanistic models.

### Text mining

Different studies show that various data mining methods can be employed to extract knowledge and detect different trends from big data such as social media data [37–43].

Text mining is a process that uses unstructured data (text) to discover intended information. Text mining techniques extract knowledge from unstructured data while data mining extracts data from structured databases. This makes it more difficult than structured data mining. Text mining can be used to discover influenza trends from social media data [23].

#### Co-occurrences analysis

Co-occurrences analysis can be used to find how frequent certain keywords are used in a document. This helps in finding related social media posts for better flu trend predictions. In addition, more analysis could be conducted using co-occurrences analysis such as medicine misuse analysis. Daniel Scanfeld et al. [44] demonstrated antibiotic misuse analysis using co-occurrences and categorization methods on social media data. Their study has also shown that social networks can be used by patients to share health information. For that reason, these kinds of networks could be used to gather knowledge to explore potential misuse of medicine. This indicates that the co-occurrences and categorization methods, along with the known flu symptoms and treatment can be used to predict flu trends in social networking sites.

#### Historical pattern analysis

Since history may repeat itself, future events can be predicted using patterns of historical events such as search queries or social media posts. Kira Radinsky et al. [45] proposed a method named PROFET that predicts future news based on patterns of historical events collected from Google trends services. These services use large number of search queries.

PROFET algorithm extracts information from large number of web resources and analyzes the past events pattern in order to predict future news. It uses Google Hot Trends, which is used to obtain the important events, and Google Related Trends for the related events. It also uses Google Trends Chart to find peaks for an event. PROFET consists of several steps:

- The algorithm identifies a set of all extracted events: *W*={*w*_1_,*w*_2_,…,*w*_*k*_}. For simplicity, only the important and related events are considered for further processes.
- The algorithm identifies a vector *D* to represent an ordered set of days: *D*=<*d*_1_,*d*_2_,…,*d*_*n*_>.
- The algorithm defines a binary vector for each event *w*_*i*_: $ g (w_{i}) = <d_{1}^{i}, d_{2}^{i}, {\ldots },d_{n}^{i}>$. This vector is used to indicate that the event *w*_*i*_ appeared when $d_{j}^{i}=1$. The Google Trends Chart is used to find peaks for each event *w*_*i*_.
- The algorithm predicts the terms or events that may peak in *k* days.
- The algorithm returns a list of candidate terms with associated weights. The event with a stronger weight is the event with a higher chance of happening in the future within *k* days.

This algorithm together with the available social media data can help in predicting flu trends in social media. The patterns of the historical social media posts can be used as an extra parameter for any machine learning framework for better predictions.

### Graph data mining

This technique is a process of discovering knowledge in structured data using graphical representation and graph theories. Courtney D. Corley et al. showed how graph based data mining can be used to discover flu affected communities and also to detect anomalies for better trend predictions [23].

**Fig. 2:**
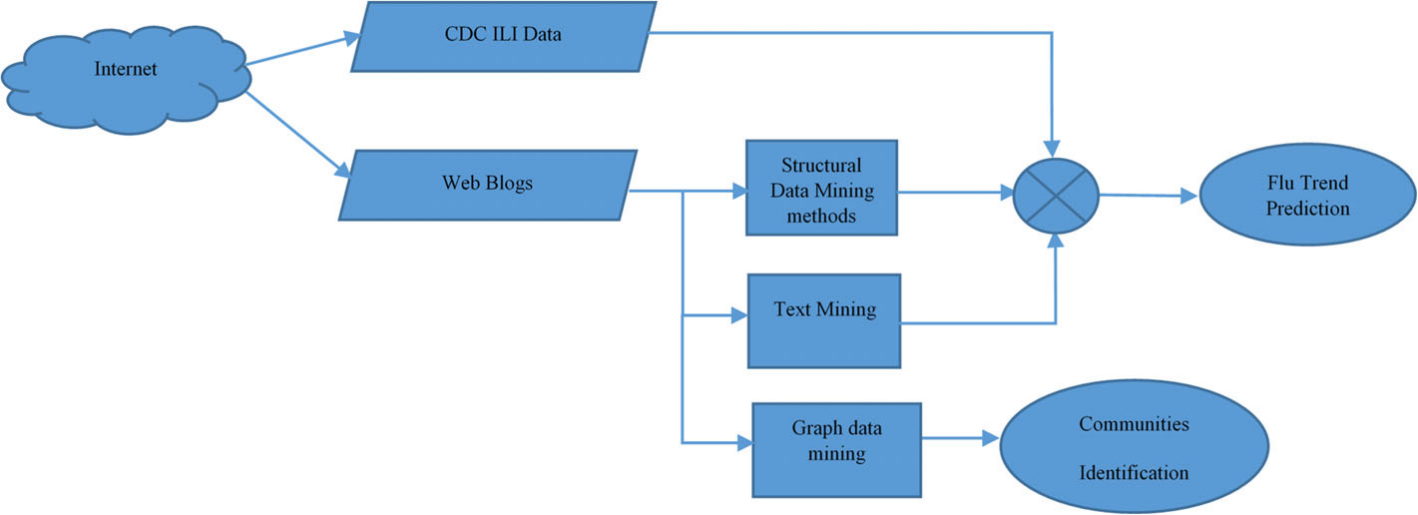
A method to monitor ILI and identify communities in Social Media. The figure shows the general overview of a proposed framework which monitors Influenza-Like Illness (ILI) mentioned in social media. It employs different data mining methods: text mining, link (graphical) mining, and structural data mining methods

Corley et al. [23] developed a framework based on text and graph mining. Figure 2 shows the general overview of their proposed framework. The framework monitors Influenza-Like Illness (ILI) mentioned in social media. It employs different data mining methods: text mining, link (graphical) mining, and structural data mining methods. The text mining method is used to identify flu trends by extracting information from large collection of texts from social media web. The link analysis is used to find the targeted communities. A community is represented as a collection of vertices and edges (V, C). The targeted community can be identified using the Girvan—Newman algorithm (GN), which helps to identify clusters of potential communities in the studied social media [23]. The clustering process in this framework is based on content type and publisher (the first responder). The graph-based analysis technique is also used for further detection of possible anomalies (unusual occurrences) and informative substructure that could increase ILI. The results of the proposed framework show high correlation between flu-related posts and CDC weekly reports. The Girvan—Newman algorithm can be applied to any graph for the clustering process. It is composed of several steps that should be iterated to identify clusters as communities. After each iteration, the remaining components in the graph are considered as a cluster/community. Finding targeted communities using this method helps in optimizing the public health responses.

### Topic models

#### Ailment topic aspect model (ATAM) and latent Dirichlet allocation models

ATAM is a topic model that associates words with their hidden topics. Michael J. Paul et al. [46] showed that the ATAM model can be used to discover health topics posted by users in Twitter. The model is designed to discover more than a single disease. It is based on a probabilistic topic model called LDA (Latent Dirichlet Allocation) that associates words to hidden topics in a text such as a Twitter post and then discovers latent (hidden) structures in the data. Each hidden topic in any document is defined by a multinomial distribution over its words. Applying posterior inference (parameter learning) will return the topics with the words, which frequently co-occur with them. LDA gives topics related to disease, but it doesn’t indicate a specific ailment clearly. For example, surgery could be discovered as a treatment, but LDA doesn’t identify clearly whether it is for an injury or cancer. In addition to the topic model, the authors developed a structural model which uses symptoms and treatments to discover ailments.

ATAM can be used to associate symptoms, treatments, and general words with an ailment (disease). An ailment comprises of treatment, symptoms and general words. The model could associate a disease with its symptoms and treatment using social networking sites. The authors use 1.6 million tweets to train the model. The model is a low cost alternative to track public health trends. It has been shown that the ATAM model can discover more ailments than LDA. It produces more detailed analysis, and it tracks disease rate which matches the statistics published by the government (CDC).

#### Enhanced topic models (ATAM+)

Paul et al. [47] proposed a variant version of ATAM model called ATAM+. It is an enhanced model that can be used based on what can be learned from Twitter for public health to predict specific diseases such as influenza among other things. The model is improved by using prior knowledge, reports resulting from several new applications, correlating behavioral risk factors with ailments, and analyzing correlation of symptoms and treatments with ailments. The improved process consists of selecting 20 diseases and then collecting articles related to these diseases based on prior knowledge, and in the second step, the words in the articles were paired with the selected diseases. The results of the improved model show high quantitative correlation with government data (CDC) in detecting the flu trend using social media.

The study shows that by using ATAM+, the following could be learned from Twitter:

- Syndromic Surveillance: ATAM+ is able to discover and learn several aspects of public health, not only flu or just specific diseases from Twitter. The correlation between the results of the improved model and flu rate produced by CDC is high (0.958).
- Geographical Behavioral Risk Factor: This shows how the model can be used to mine public health information based on geographical region. In comparison with the ATAM model, it has been shown that the ailments discovered by the enhanced model (ATAM+) have higher correlation with the risk factors run by CDC. For example, the correlation between cancer and tobacco use is (0.648) using ATAM+ whereas the correlation is (0.320) using ATAM. This demonstrates that the ATAM+ outperforms ATAM.
- Ailment Tracking over Time and Geography: ATAM+ model can be used to mine data over time and different locations.
- Symptoms and Medication Analysis: The analysis of symptoms and treatment -especially for people who don’t go to health care providers - needs a large population sample size. Therefore, SNS is a better alternative to perform symptoms and treatment analysis using ATAM+. The ATAM+ is able to detect that the headache is the most common ailment treated by pain relievers. Also it shows that Tylenol is the most popular pain reliever on the market.
- Antibiotic usage Analysis: Medicine usage analysis such as antibiotic misuse could be performed using ATAM+.

#### Hidden flu-state from tweet model – HFSTM (users health states transition for better prediction)

Liangzhe Chen et al. [48] proposed a model called Hidden Flu-State from Tweet Model (HFSTM) that is able to capture hidden health states of users and the associated transitions by analyzing their tweet posts. The extracted states are used to obtain better prediction of trends. It aggregates the states of the users in a specific geographical region for better prediction. The proposed model captures not only one tweet post, but also streams of tweet posts of users in order to capture their underlining health status (different health states from tweet posts). The used states for this study are: S (healthy), E (Exposed), I (Infected), and R (Recovered with Immunity).

Most of the other models are coarse-grained because they don’t give any understanding of how health states change over time. This model links between the social activity models and the epidemiological models. This linkage improves the prediction process. The most common Contagion-based epidemiological models are SI, SIR, SEIS. These models are used here to predict the true flu cases by tracking the health states of a person through the lifecycle of the infection.

Unlike the proposed model, the existing topic models (LDA, ATAM+, Makovian, and non-Markov) don’t solve the problem of flu state changing. The model uses unsupervised topic modeling which can capture the transition (changes) between consecutive messages of a user.

**Fig. 3:**
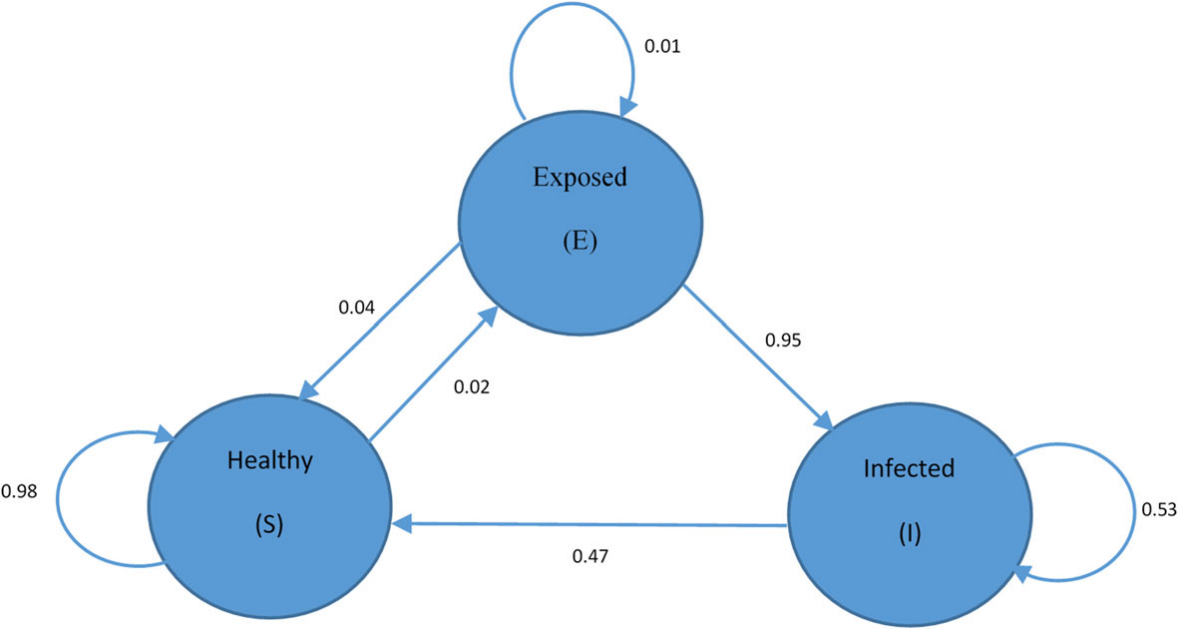
Health state transition diagram. The figure shows that the HFSTM model could learn the state transition between the three states (S, E, I)

The study shows that the HFSTM model could learn meaningful word distribution. Each word in the list belongs to one of the three states (S, E, I). It can also learn the state transition as shown in Fig. 3. The HFSTM model is able to classify the state of tweets and captures the transitions. It is also capable of predicting flu trends. The results of HFSTM model were compared with the Pan American Health Organization (PAHO) weekly records and the results of other two models: Google Flu Trend (GFT) and the baseline model that is based on word count and linear regression. GFT is a Flu trend prediction system which uses the volume of flu related search queries for the prediction process. Many studies have been conducted to evaluate and improve GFT [49–54]. It has been shown that the HFSTM model is better than the baseline model and is comparable with GFT. In some cases, HFSTM outperforms GFT. Results have shown that GFT overestimates the number of flu cases.

### Machine learning techniques

#### Support vector machine (SVM)

Support Vector Machine (SVM) is a supervised learning method. Based on our survey, SVM is the most commonly used machine learning algorithm for the purpose of flu related posts classifications [55–59].

David A. Broniatowski et al. [57] proposed a model that consists of three levels of classification using SVM for better distinction between the actual tweets about flu and the tweets that look related but are not actually flu tweets (named “chatter” posts). The first classifiers is used to classify the collected posts to health-related/unrelated posts. The second one is used to extract the flu related posts and the third one is used for infection classifications. The proposed algorithm was tested using a collection of tweets from Sep. 30, 2012 to May 31, 2013 (covering the season flu of 2012-2013) for the NYC location and USA in general (local and national). To measure the performance, the results of the proposed algorithm was observed to have correlated with the CDC data (*r*=0.93) and also with the data of the Department of Health and Mental Hygiene of New York City (*r*=0.88).

It has been shown that the distinction between the infection and awareness tweets enhances the accuracy of the results. The goal of this distinction is to consider only the infection posts. Alex Lamb et al. [60] proposed a machine learning based model that consists of two phases of classification to differentiate between the infection and awareness tweets. The accuracy of the model showed high correlation with CDC data using Pearson correlation (*r*=0.9897).

Eiji Aramaki et al. [56] proposed a framework that consists of two parts. First, a crawler which works together with Twitter API to collect tweets was used and then they were filtered for only flu-related ones. Second, an SVM-based classifier was used to extract only the actual influenza tweets (positive tweets) and exclude the unrelated ones such as news and questions (negative tweets). The initial dataset for this study was collected from Nov 2008 to June 2010. It included 300 million general tweets. Then, this dataset was filtered using “Influenza” keyword to get a set of only flu related tweets which contained 400,000 tweets. The flu-related dataset was divided into two parts: a training dataset which contained 5000 tweets (November 2008) and a test dataset which contained all the remaining tweets from Dec 2008 to June 2010. The training dataset was assigned to a human annotator to label each tweet for being either positive or negative. A tweet is labeled positive if it met two conditions. First, the flu tweet should be about the person who posted the tweet or about another person in a nearby area (maximum an area of the city). If the distance is unknown, the tweet is considered negative. Second, the flu tweet should be an affirmative sentence and in a present tense or past tense with maximum period of 24 hours which can be checked using specific keywords such as “yesterday”. The SVM classifier was implemented using the Bag-of-Words feature representation. The authors compared the accuracy of the SVM-based classifier with other 6 different machine learning methods and they found that the SVM was the most accurate method. For the purpose of evaluation, a Pearson Correlation was used to correlate between the results of this framework and the Japanese government data provided by the Infection Disease Surveillance Center (IDSC). The results of this framework showed high correlation (*r*=0.89). The results also showed that news could impact the accuracy of the results. It has been shown that the swine flu related news in 2009 led to poor performance of this method and other methods.

José Carlos Santos et al. [59] also applied SVM-based classifier to detect flu-like illness in Portugal using twitter posts. For the purpose of training and testing, a dataset with 2704 posts was manually annotated with 650 textual features. A subset of the annotated dataset was used to train the classifier. The classified tweets together with search queries were applied to a regression model as predictors. The results of the used model was evaluated and compared with the reports provided by Influenzanet: a system that monitors Influenza Like Illness activities in Europe. The highest correlation ratio between the results of this method and Influenzanet data is 0.89 (*r*=0.89). The classifier was implemented using the Bag-of-Words feature representation and the feature selection process was based on a Mutual Information (MI) value which is used to pick the best set of features. Each feature is applied to a true class and then MI value is assigned to the feature. The value of MI is based on how the feature is related to the true class. A feature with high MI value is more related to the true class.

Nanhai Yang et al. [58] proposed a SVM-based method to predict flu trends from Chinese social networking sites in Beijing. Authors claim that this is the first study to predict flu trend from Chinese social networking sites. The collected data for this study included 3,505,110 posts from Sep. 2013 to Dec. 2013. Among those, 5000 random posts were selected for manual annotation (sick and not sick labels) to be used for training and testing purposes. Two hundred eighty five of sick posts and 285 of not sick posts were picked for training. For higher accuracy, word based features were used instead of character based features. Among the four types of word weighting: Boolean weighting, term frequency weighting (TF), inverted document frequency weighting (IDF) and term frequency-inverted document frequency weighting (TFIDF), the TFIDF method was considered for classification purposes. Different classifiers were compared to decide the best for the problem. Authors found that SVM was the best for big data problems. This method was able to predict the flu trend five days earlier than the China Nation Influenza Center (CNIC).

Mauricio Santillana et al. [55] proposed a machine learning-based method that was capable of predicting flu related activities. In addition to CDC ILI reports that have been used as ground truth, the method used data from different sources for better results. The sources included Google searches, Google Flu Trends, Twitter posts, hospital visits records collected from AthenaHealth, and a surveillance system called FluNearYou. This study has shown that the results of prediction methods using combined data sources outperform the results when using a single data source. The method utilizes well-known machine learning algorithms including support vector machine, stacked linear regression and AdaBoost with decision trees regression. The study has also shown that the three algorithms work perfectly together in combining the information from different sources for real time analysis and then better forecasting. It has been shown that this method can predict one week faster than the Google Flu Trend (GFT) with accurate and comparable results.

#### Neural network

Vasileios Lampos et al. [61] proposed a method to track flu in the population using social networking sites. The method analyzed flu-related and flu-symptoms-related keywords in Twitter. The extracted information was converted to flu-score using machine learning techniques. Computing the flu score from Twitter includes several steps. First, a set of selected keywords *M* is identified to represent the search keywords to look for in Twitter posts: *m*_*i*_; where *i*∈ [1,*k*]. Second, a set of daily tweets is identified as *τ*=*t*_*j*_ where *j*∈ [1,*n*]. When the marker *m*_*i*_ appears in the tweet *t*_*j*_:*m*_*i*_(*t*_*j*_)=1, otherwise *m*_*i*_(*t*_*j*_)=0. The number of markers appeared in *t*_*j*_ divided by the total number of markers is denoted as *s*(*t*_*j*_) and calculated using Eq. 1.

1 $$ {S (t_{j})= \frac {\sum_ i m_{i}(t_{j})}{k}}  $$

The flu-score of the daily tweet corpus *f*(*τ*,*M*) equals to the sum of all the flu-score of the tweets *s*(*t*_*j*_) of that day divided by the total number of the tweets *n*.

2 $$ f (\tau,M)= \frac {\sum_ j s(t_{j})}{n}= \frac {\sum_ j \sum_{i} m_{i}(t_{j})}{k\times n}  $$

An extension was made to the previous model in order to make better prediction of Health Protection Agency (HPA) flu rate by adding weight *w*_*i*_ to each marker *m*_*i*_. Therefore, the weighted flu-score for each tweet is:

3 $$ S_{w} (t_{j})= \frac {\sum_ i w_{i}\times m_{i}(t_{j})}{k}  $$

Then, the weighted flu scores of all tweets of a day is summed up to get the weighted flu-score of the daily tweet corpus *f*_*w*_(*τ*,*M*):

4 $$ f_{w} (\tau,M)= \frac {\sum_ j s_{w}(t_{j})}{n}= \frac {\sum_ j \sum_{i} w_{\times} m_{i}(t_{j})}{k\times n}  $$

The contribution of the marker *m*_*i*_ in the daily tweet flu-score *f*_*w*_ is considered as flu-subscore $f_{(w_{i})} (\tau,m_{i})$:

5 $$ f_{w_{i}}(\tau,m_{i})= w_{i} \times \frac {\sum_ j m_{i}(t_{j})}{k\times n}  $$

Using the flu-subscore $ f_{w_{i}} (\tau,m_{i})$, the daily tweet flu-score could be represented as a vector of flu-subscore *F*_*w*_ of all the markers (keywords):

6 $$ F_{w} = \left[f_{w_{1}} (\tau,m_{i}),......., f_{w_{k}} (\tau,m_{k})\right]^{T}  $$

The weights *w*_*i*_ of markers *m*_*i*_ can be learned by:

1. Initially, the unweighted flu-score vector *F*_*w*_ that is the sum of unweighted flu-subscore smoothed with 7-point moving average is found.
  7 $$ F = \left[f (\tau,m_{1}),......., f (\tau,m_{k})\right]^{T}  $$
2. The least square linear regression between *F* from the smoothed version, *F* from the expanded one, and smoothed HPA flu rate is performed.

To maximize the correlation with HPA flu rate, Vasileios Lampos et al. [61] also proposed a method to extract the markers (keywords) automatically. This method consisted of two steps. First, a list of candidates was created by extracting them from trusted web documents related to influenza. Second, the most informative ones were picked using the Least Absolute Shrinkage and Selection Operator (LASSO) method that discards the redundant features of the candidates. The use of LASSO method is explained in detail in [61].

**Fig. 4:**
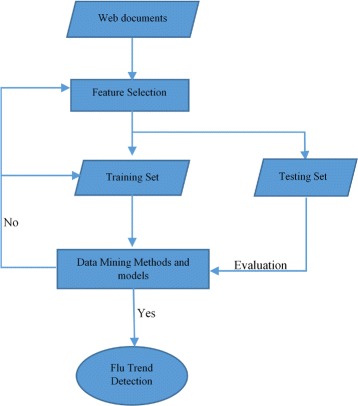
A framework for influenza outbreak detection. The figure shows a framework of a model to detect influenza outbreaks by analyzing web search queries using a neural network approach

**Fig. 5:**
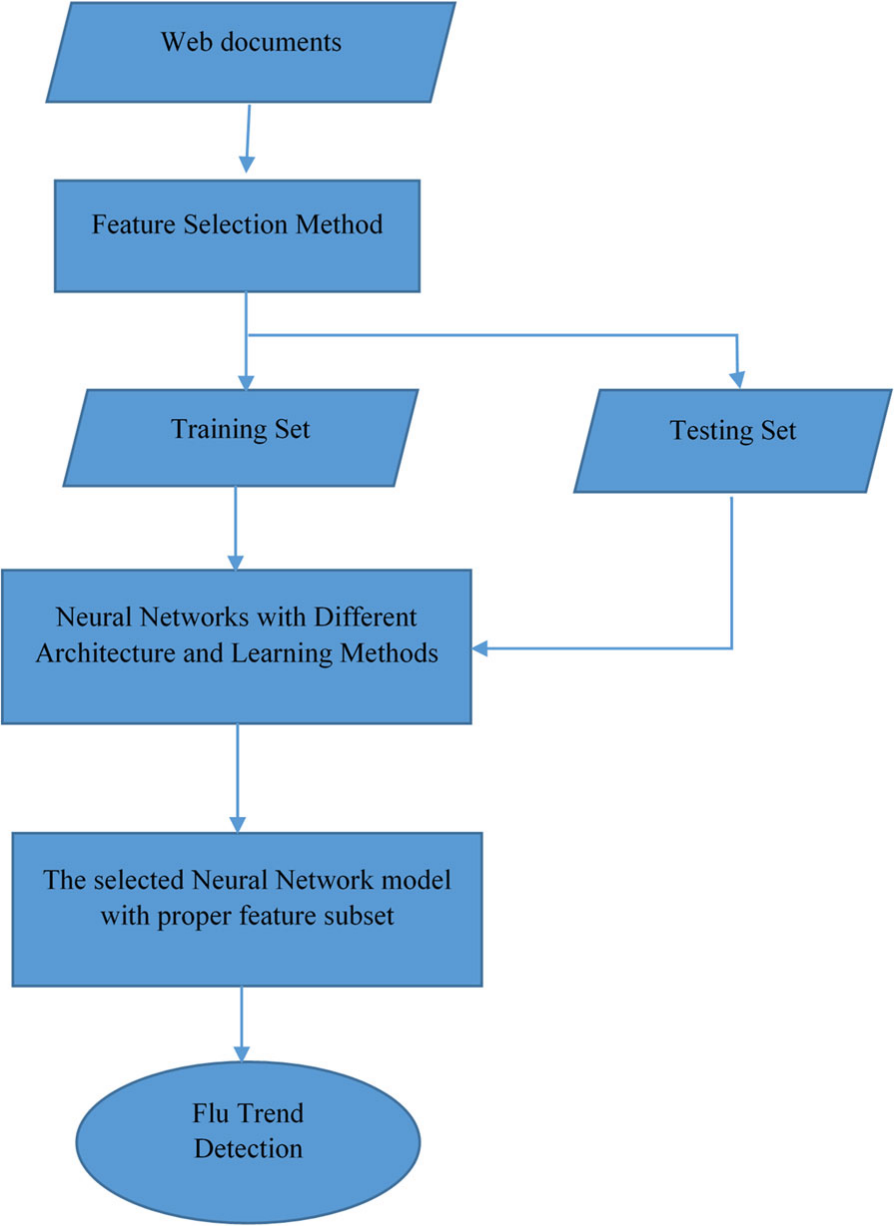
The process of Neural Networks based detection. The figure shows an overview of the steps of the detection model based on Neural Networks

Another machine learning technique that can be used in early trend prediction is neural network. Disease outbreaks can be predicted using Neural Network (NN) based approaches to analyze web data. Wei Xu et al. [62] proposed a model to detect influenza outbreaks by analyzing web search queries using a neural network approach. Figures 4 and 5 show an overview of their proposed approach. It consists of several steps. The first step is to collect data from search engine queries and ILI data from the CDC. The second step is to select features automatically by reducing the dimension of the query and keeping only the most important features. The third step is to find the relationship between the Influenza Like Illness (ILI) and web data (query data) using different NN with different algorithms and architectures to measure the fitness values. The NN used with this model are: NN-GDX (Gradient descent with momentum and adaptive learning rate back propagation), NN-OSS (One-step secant back propagation), and NN-RP (Resilient back propagation). The 10-fold cross validation method is used to validate the different NN algorithms. The fourth step is to select the best NN as a detector using the cross validation method. The fifth step is to use the selected NN (detector) with the best features subset to predict flu activities. The accuracy (ACC) of the results of each NN is measured using Eq. 8. If *A*_*i*_ are the actual values, *D*_*i*_ the detection values, and *N* the number of given pairs (*A*_*i*_,*D*_*i*_), then

8 $$ ACC= \frac {1}{N} \sum_{i=1}^{N} \frac {D_{i}}{A_{i}}  $$

Results show that NN-RP was the best to be used for influenza detection. NN-RP had the best average of ACC values.

#### Naive Bayes

Kenny Byrd et al. [63] proposed a framework based on Naïve Bayes classifier. The framework consisted of several steps. The first step was tweets collection with a location filter. The collected tweets were from Oct. 27 to Nov. 30 of 2015. The dataset included a total of 1,848,130 tweets. The used location filter was provided as latitudes and longitudes pairs (comma separated list) to specify a bounding box of a required area. The Google Maps Developer tool was used to determine the bounding boxes of the required areas (cities). For this study, the used location was the area of Ottawa and its surrounding areas. The second step was flu-related tweets filtration. The used keywords for the filtration process were “sick”, “flu” and “cough”. The total of filtered tweets were 4696 posts. The third step was pre-processing which included: stop words elimination, URL’s removing, words stemming, and retweets removing. The fourth step was sentiment analysis by applying machine learning techniques for classification (positive, negative, neutral). Three machine learning algorithms were evaluated and it was found that the highest accuracy method was the Naïve Bayes classifier. The Naive Bayes classifier was implemented using the Stanforn core NLP (Natural Language Processing) and trained using the OpenNLP training dataset which includes 100 annotated tweets. The sentiment analysis is considered accurate when there is matching between the predicted sentiment polarity with the manual assigned opinion of the sentiment. Authors found that Naive Bayes was the most accurate one with 70% matching.

#### Prediction market using support vector machine regression algorithm (SVR)

The prediction market is a mechanism that can be used for future prediction based on creating ’shares’ for an event. People can trade these shares with prices determined by the market. The prices can be used as probability of the event occurrence. This is considered as one of the optimal prediction solutions, and it is less expensive than other prediction methods. Disease outbreak can be predicted using the prediction market together with the Support Vector Machine regression algorithm (SVR) using share prices [64]. Joshua Ritterman et al. [64] have shown that the prediction of swine flu in 2009 was more accurate when adding some features extracted from social networking sites to the SVR. The prediction market is modeled in two different ways: internal market and external market.

##### Internal market

The internal market is based on time series. It uses historical prices for today’s price prediction. Technically, the prediction for a given day *F*_*n*_ is achieved by using the average price of the previous day *AvgP*_*n*−1_ divided by the sum of the average prices for the previous 5 days (Eq. 9).

9 $$ F_{n} = \frac {Avg P_{n-1}}{\sum_{i=2}^{6} Avg P_{n-i}}  $$

The SVR is trained using extra features. The first feature is to use the Short-Term history feature *F*(*n*)=*AvgP*(*n*−1) that is the average price of the previous day. It gives a quick overview of the price movement. The second feature is the Mid-Term history feature that is the moving average price, calculated using Eq. 9. This gives a longer period than the first feature. The third extra feature is the Long-Term feature that is the sum of a vector of binary values *M*, as shown in Eq. 10. The Long–Term feature is used to indicate the market direction for a long time.

10 $$ F (n) = \sum_{i=0}^{n-1} M_{i}, M_{i} = \left\{ \begin{array}{c} M_{i-1} + 1\ \text{if}\ Avg (P_{i}) \ge Avg (P_{i-1}) \\ M_{i-1} - 1\ \text{if}\ Avg (P_{i}) < Avg (P_{i-1})\\ \end{array} \right.  $$

##### External market

This way of modeling considers the fundamental products of the company and the events occurring around the world. The SVR classifier is trained using social media data. By using the social media data, SVR is trained with unigram and bigram and their frequencies using social media data (i.e. daily counts of unigram and bigrams). No internal market is given for training. This gave lower performance compared to training with only a subset of data. For better performance, the system should be trained with only relevant data. This can be accomplished by training the SVR with unigram and bigram for a specific period of time based on historical context provided to the system. The length of the period is decided by the system using the historical context to determine the news cycle.

It has been shown that combining the prediction market with features extracted from social networking sites leads to better results. This demonstrates that social media data played an important role in the 2009 swine flu trend prediction.

### Math/statistical based models

#### Autocorrelation function (ACF)

ACF finds the correlation of the values of the same variables at different times (*x*_*i*_,*x*_(*i*+1)_). Therefore, this method can be used for disease outbreak predictions. Disease outbreak trends in social networking sites can be monitored by tracking a sudden high frequency of disease-content posts using ACF. It compares the averaged disease-related posts per day with the actual number of the same disease posts of that day. Courtney D Corley et al. [65] proposed a method to track ILI in social media using ACF and to identify possible web and social media communities [65]. The method tracks a sudden high frequency of flu-content posts using ACF. The method defines a seven day period as a period cycle for better accuracy and anomaly detection. It starts on Sundays and ends on Saturdays.

The results of this methodology showed strong correlation with CDC reports. The Pearson correlation coefficient is used for evaluation. The value of *r* was 0.767 with a confidence level of 95%.

Web Social Media (WSM) community identification and analysis was used as a part of their methodology for better results by using link analysis. Link analysis was also used to identify the first responder or influential user of a community. Only the links between flu posts are considered. The links between a flu-related post and non-flu-related post are not considered in the defined community. Closeness, Betweenness and Page Rank measures were used to rank flu communities to tell how a blog’s influence disseminates flu information. Blogs with high closeness and page rank can spread flu-information (response) more quickly.

##### Closeness

It is used to find the average of the shortest paths between actor *v* and the other reachable actors. It is defined as shown in Eq. 11 [66]. Let *i* and *j* be actors, *d*(*i*,*j*) be the distance function that finds the number of geodesics between *i* and *j*, and $\sum _{j=1}^{N} d(i,j)$ be the total distance of *i* from all other actors. Closeness is defined as follows:

11 $$ C_{c} (i)= \left[\sum_{j=1}^{N} d(i,j)\right]^{-1}  $$

##### Betweenness

It measures how a blog is central among other blogs. It is defined as shown in Eq. 16 [66]. Let *g*_*jk*_ be the number of geodesics between *j* and *k*, and *g*_*jk*_(*i*) be the number of geodesics between *j* and *k* that contain actor *i*. Betweenness is defined by the following formula:

12 $$ C_{B} (i)= \sum_{j<k} \frac {g_{jk}(i)}{g_{jk}}  $$

##### Page rank

It is an eigenvector centrality which measures the importance of a node. It is defined as shown in Eq. 13 [65]. Let *d*=0.85 be a factor, where the pages are represented using the symbol *P*_*n*_, the set of pages linked to *P*_*n*_ is represented using *M*(*p*_*n*_), and the out links on page *P*_*j*_ is represented using *L*(*p*_*j*_). Page Rank relationship is shown as follows:

13 $$ R_{p_{n}} = \frac {1-d}{N} + d \sum_{pj\in M (p_{n})} \frac {PR(p_{j})}{L(P_{j})}  $$

#### Auto regression moving average (ARMA) / SNEFT framework

ARMA is a stochastic model which is composed of two forms: Auto Regression (AR) model and Moving Average (MA) model. The AR model is a prediction model. Its output depends linearly on the past values, a random value as an error, and a constant value. The MA model is used to represent the correlation between the past values and the white noise using linear regression.

**Fig. 6:**
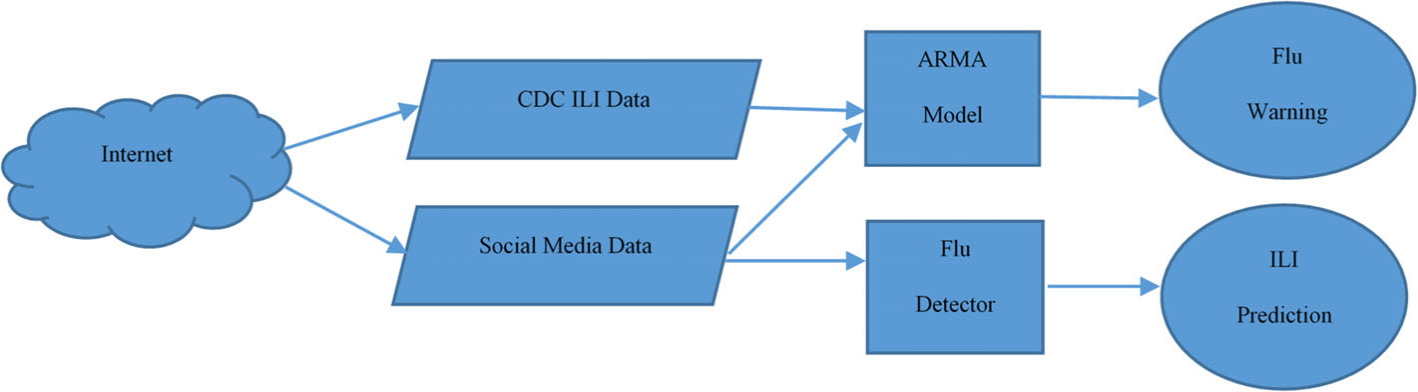
SNEFT architecture. The figure shows the architecture of the SNEFT framework. It utilizes the ARMA model and the data obtained from CDC. Both are used in collaboration for better flu prediction trends

Based on the ARMA model, Harshvardhan Achreckar et al. [67] proposed a framework called Social Network Enabled Flu Trends (SNEFT) that utilizes the ARMA model and the data obtained from CDC. Both are used in collaboration for better flu prediction trends. The architecture of the SNEFT framework is shown in Fig. 6. The architecture consists of two main parts. The first part is used to predict influenza and Influenza Like Illness (ILI) using CDC data. The second part is used to provide flu warnings using Twitter data. The Auto regression Moving Average (ARMA) model is used to predict ILI incidence as a linear function of current and old Social Network data and historical ILI data (CDC data). The results showed that Twitter data improved the output of the statistical models that were used for prediction. The SNEFT framework was tested with and without Twitter data together with CDC reports. It has been found that the Twitter data improved the accuracy of the prediction model. Based on their findings, it is clear that Twitter could provide real time measurement of influenza activity in the population.

#### Numerical-based analysis

Sangeeta Grover et al. [68] proposed a framework to detect flu outbreak with respect to three stages of epidemics (beginning of epidemic, spread of epidemic, absence of epidemic) using the Bag-Of-Words (BOW) technique. The BOW is a technique that learns a vocabulary from all of the documents, then models each document by counting the number of times each word appears. The implementation of this framework consists of the following steps:

- Collect tweets using twitter API.
- Store the collected tweets in MangoDB.
- Build Bag-Of-Words (BOW) for each stage of epidemic (beginning of epidemic, spread of epidemic, absence of epidemic)
- Apply the Swine Epidemic Hint Algorithm (SEHA) on the tweets. The text of a tweet is tokenized for numerical analysis. The numerical analysis checks how relevant the tweet is to the epidemic stages.
- Classify the tweets into the 3 stages of the epidemics. The classification process is based on the numerical results from the previous step.
- Evaluate the results of this framework using 6 cross validation of Gaussian regression and prediction model. The results show that the framework was fairly accurate since the average value of the error rate was about 1.1.

### Mechanistic disease models

Mechanistic disease models are used to provide a better understanding of any epidemic dynamics. Unlike statistical models, the mechanistic models consider different features to estimate key epidemic parameters such as intensity and severity that impact public health decision responses [69, 70]. Within the various mechanistic models, metapopulation models, compartmental models, and agent-based models provide information on population epidemic states and individual progress of an epidemic.

#### Metapopulation models

Metapopulation models, such as Global Epidemic and Mobility (GLEAM) model, are spatial, stochastic and individual based models that can simulate the spread of epidemic diseases at worldwide scale. The model divides the world into smaller regions defining subpopulation networks and connections between the subpopulation which represent the individual fluxes due to the transportation and mobility infrastructure [71].

Qian Zhang et al. [70] proposed a seasonal flu forecasting framework based on mechanistic disease model (GLEAM). The framework was validated and tested by comparing the results from the framework with the official government data in the US, Italy and Spain in the 2014-2015 season and 2015-2016 season. The framework is a combination of the social media data, official surveillance data and mechanistic modeling approach. It consists of three stages. In the first stage, data from official surveillance systems and Twitter is used for model initialization. A set of English ILI-related tweets for a given region is used as an initial condition of relative flu incidences and as an input for the framework. The data from official surveillance systems is used to evaluate the coefficient of determination of the used ILI search keywords. The second stage consists of exploring important parameters: population, infectious period and the effective reproduction number (number of infected individuals in a region). The third stage is parameter selection and prediction. It has been shown that the framework provides reliable results for epidemic intensity and peak timing up to 6 weeks in advance. The accuracy of the framework showed high correlation with official surveillance data using Pearson correlation (the highest *r* value is 0.98 for the flu prediction with one week in advance).

#### Compartmental models

Compartmental models define the rate at which individuals move between defined compartments and divide the population into subpopulation based on disease states. Examples include susceptible–infectious–recovered (SIR) and Susceptible-Infections-Recovered-Susceptible (SIRS) [72].

Liangzhe Chen et al. [48] proposed a model called Hidden Flu-State from Tweet Model (HFSTM) based on the concept of epidemiological compartmental models. It analyzes a stream of a user’s tweets and captures the disease states and the associated transitions.

Jeffrey Shaman et al. [35] proposed a framework that predicts a seasonal flu using the compartmental model (SIRS) along with common used techniques in numerical weather predictions. Epidemic disease dynamics are nonlinear which are similar to weather dynamics. The nonlinearity of the epidemics makes the prediction systems sensitive to the initial and current conditions. Like any nonlinear system, it is possible that the error rate of the system will grow with further uses which leads to inaccurate results. To overcome the growth of error rates with the non linear systems, data assimilation techniques such as filtering are used to update and adjust the system using the latest available observations. The applied data assimilation method in the presented framework is the Ensemble Adjustment Kalman Filter (EAKF) method for the updating process using weekly observations obtained from Google Flu Trend (GFT). This method combines the weekly GFT observations into the Susceptible Infections Recovered Susceptible (SIRS) model. The EAKF is a recursive filtering technique to estimate the state of the model using a combination of the observations and the evolving ensemble of the model simulations. The framework was validated and then used to perform simulation of influenza prediction in the New York city for the 2004-2005 and 2007-2008 flu seasons. It has been shown that the framework is able to predict the peak timing up to 7 weeks in advance.

#### Agent-based models

Agent-based models define entities (agents) that interact with each other and the surrounding environment based on specific rules. These models provide better understanding of the change of individual behaviors during an epidemic which help in outbreak predictions [72].

Suruchi Deodhar et al. [73] developed a large scale web application called FluCaster for flu epidemic forecasting using agent-based models. This model can distinguish FluCaster from other available systems. It produces fine-grained results that helps decision makers in performing detailed analysis. For example, filtering the results of the flu forecast by a specific location for a specific age sub-population in a specific time can be provided by this model. FluCaster was implemented using CDC surveillance data and Google Flu Trend (GFT).

### Detection based on filtered keywords and documents

Simple flu related keywords can be used to produce accurate results with a high correlation with CDC weekly reports. The method of selecting search keywords is very important. It impacts the accuracy of the results. Selecting keywords based on correlation with national statistics may cause inaccurate results. For example, the “flu shot” term has a high correlation but it does not necessarily reflect the spread of flu. It could be just a general discussion about it or an advertisement. Therefore, a document classifier to remove spurious matches (such as advertisements) can be used to get more accurate results and reduce the error rates [74]. Aron Culotta [74] presented a method of correlating the keywords with ILI rates from CDC. Let *P* be the ILI symptoms reported by providers, *W*={*w*_1_,*w*_2_,...,*w*_*k*_} be the set of keywords, *D* be a document collection, *D*_*w*_ be a set of documents that at least contain a keyword in *W*, *B*_1_ and *B*_2_ be coefficients, *e* be error terms, and *Q*(*w*,*D*)=|*D*_*w*_|/|*D*| be a query fraction, then

14 $$ \log (P) = B_{1} (log (Q (w,D)) + B_{2} + e  $$

Removing spurious keywords such as a keyword within government announcements and advertisements may also help produce better results and improve the correlation with ILI reports. Aron Culotta [74] also proposed a document classifier that can be used for document filtration. It labels the messages as ILI related or not. Then, the classifier calculates the probability of the ILI reporting messages. This classifier should be trained using logistic regression with parameter *θ* that can be computed using the limited memory quasi-Newton method for large scale optimization (L-BFGS). Details of the L-BFGS method and its implementation is discussed in [75]. Let *y*_*i*_ be a binary random variable where (1) is a positive document and (0) otherwise, *x*_*i*_={*x*_*ij*_} be a vector of random values where *x*_*ij*_ is the number of times word *j* appears in document *i*, *D* be a document collection, *θ* can be computed using L-BFGS gradient descent [75]

15 $$ P\left(y_{i} = 1| x_{i};\theta\right) = \frac {1}{1+e^{\left(-x_{i}.\theta\right)}}  $$

The filtration process was combined with regression in Eq. 14 by considering two kinds of classifying methods: soft classification and hard classification. The soft classification finds *Q*_*s*_(*W*,*D*) of positive documents using Eq. 16. This method assigns the probability as a weight to each matched document in *D*_*w*_. The hard classification finds *Q*_*h*_(*W*,*D*) by considering and counting only the documents with probability of positive class >0.5 using Eq. 17. Afterwards, the value *Q*(*w*,*D*) is substituted in Eq. 14.

16 $$\begin{array}{*{20}l} Q_{s} (W,D)&= \frac{\sum_{d_{i}\in D_{w}} P\left(y_{i} = 1| x_{i};\theta\right)}{|D|} \end{array} $$

17 $$\begin{array}{*{20}l} Q_{h} (W,D)&= \frac{\sum_{d_{i}\in D_{w}} \left(P(y_{i} = 1| x_{i};\theta)>0.5\right)}{|D|} \end{array} $$

The results show strong correlation for most of the picked keywords (e.g. flu, cough, sore throat, and headache). Comparing the results with another study’s results by Lampose and Christianini (2010) [61] has shown that the results are competitive and yield less complexity. This concludes that flu trends could be predicted in a population by using simple methods.

## Discussion

A summary of the used data sets in the reviewed studies is shown in Table 1. The performance of the discussed methods is shown in Table 2. Most studies use Pearson correlation and Root Mean Squared Error (RMSE) for performance measurement. Therefore, in Table 2, the Person correlation measure is included for comparison.

**Table 1:** Summary of the used data sets in the reviewed studies

Method category	Method name	Study reference	SNS	Language	Timeframe	Location
Graph data mining	Graph data mining	[23]	Twitter	English	Oct 2008 - March 2009	US
Text mining	Historical patterns	[45]				
	Co-occurrences	[44]	Twitter	English	March 2009 - Jul 2009	
Topic models	ATAM	[46]	Twitter	English	May 2009 - Oct 2010	US
	ATAM+	[47]	Twitter	English	May 2009 - Oct 2010	US
	HFSTM	[48]	Twitter		Dec 2012 - Jan 2014	South America
Machine learning	Neural network	[61]	Twitter		Jun 2009 - Dec 2009	UK
	SVM	[57]	Twitter	English	Sep 2012 - May 2013	US
		[56]	Twitter		Nov 2008 - Jun 2010	Japan
		[59]	Twitter	Portuguese	March 2010 - Feb 2012	Portugal
		[58]	Chinese Sina	Chinese	Sep 2013 - Dec 2013	China
		[60]	Twitter	English	May 2009 - Oct 2010	US
		[55]	Twitter	English	Nov 2011 - Feb 2015	US
	Prediction market using SVR	[64]	Twitter		April 2009 - Jun 2009	
	Naive Bayes	[63]	Twitter	English	Oct 2015 - Nov 2015	Ottawa
Math/Statistical models	SNEFT	[67]	Twitter		Oct 2009 - Oct 2010	
	ACF	[65]	Twitter	English	Aug 2008 - Sep 2008	
	Numerical-based analysis (SEHA using BOW)	[68]				
Mechanistic disease models	Metpopulation model	[70]	Twitter		2014-2015, 2015-2016	US, Spain, Italy
	Compartmental model	[35]	Twitter		Dec 2012 - Jan 2014	South America
	Agent-based model	[73]				
Keys/Documents filtration	Keys/Documents filtration	[74]	Twitter	English	Sep 2009 - May 2010	US

**Table 2:** Summary of the reviewed methods and techniques

Method category	Method name	Study reference	Performance metric	Metric value
Graph data mining	Graph data Mining	[23]	Pearson correlation	*r* = 0.545
Text mining	Historical patterns	[45]	The precision for 1-day prediction is 0.8 (with mean of 0.52) and 0.6 (with mean of 0.46) for 7-days prediction.	
	Co-occurrences	[44]		
Topic models	ATAM	[46]	Pearson correlation	*r* = 0.934
	ATAM+	[47]	Pearson correlation	*r* = 0.958
	HFSTM	[48]	Mean square error (MSE)	MSE = 40.67
Machine learning	Neural network	[61]	ACC (Eq. 8)	ACC = 0.9532
	SVM	[57]	Pearson correlation	*r* = 0.93
		[56]	Pearson correlation	*r* = 0.89
		[59]	Pearson correlation	*r* = 0.89
		[58]		
		[60]	Pearson correlation	*r* = 0.9897
		[55]		
	Prediction Market using SVR	[64]		
	Naive Bayes	[63]	Sentiment polarity is used to determine the accuracy of the used method (Naive Bayes polarity is 70%)	
Math/Statistical based models	SNEFT	[67]	Pearson correlation	*r* = 0.9846
	ACF	[65]	Pearson correlation	*r* = 0.767
	Numerical-based analysis (SEHA using BOW)	[68]	RMSE	Avg (RMSE) = 1.1
Mechanistic disease models	Metpopulation model	[70]	Pearson correlation	*r* = 0.98
	Compartmental model	[35]		
	Agent-based model	[73]		
Keys/Documents filtration	Keys/Documents filtration	[74]		

Pearson correlation is a metric that evaluates the correlation between two datasets using the symbol *r* that ranges between (1) and (-1): the value of *r*=1 when both datasets exactly match and the value of *r*=0 when there is no correlation between the two datasets. Let *y*_*i*_ be the observed value of the ground truth (CDC ILINet data), *x*_*i*_ be the predicted value by a proposed model, and $\overline {y}$ and $\overline {x}$ be the average values of {*y*_*i*_} and {*x*_*i*_}, respectively. Using these notations, Pearson Correlation value *r* is defined as shown in Eq. 18 [55].

18 $$ r = \frac{\sum_{i=1}^{n}\left(y_{i}-\overline{y}\right)\left(x_{i}-\overline{x}\right)}{\sqrt {\sum_{i=1}^{n} \left(y_{i}-\overline{y}\right)^{2}} \sqrt {\sum_{i=1}^{n} \left(x_{i}-\overline{x}\right)^{2}}}  $$

Root Mean Squared Error (*RMSE*) is an evaluation metric that provides an indicator of comparison between predicted and real values. Lower value of *RMSE* indicates more accurate results of the used model and less errors. Using the same notations for Pearson Correlation, the *RMSE* value is defined as shown in Eq. 19 [55].

19 $$ RMSE = \sqrt{\frac{1}{n}\sum_{i=1}^{n}\left(y_{i}-x_{i}\right)^{2}}  $$

As shown in Table 2, the SNEFT yields a very high correlation coefficient with the used ground truth (0.9846). It has been shown in [67] that the best results is obtained when the dataset is filtered to not include redundant posts (retweet) as well as posts from the same user within one week. In addition, the authors use Root Mean Squared Error (RMSE) to evaluate the accuracy of SNEFT. It has been found that the value of RMSE of the same filtered dataset is 0.318. Further enhancement of the accuracy can be achieved by considering only the tweets about infection as shown in [60]. The distinction between the infection and awareness tweets shows high correlation with CDC data using Pearson correlation (*r*=0.9897). The other methods were evaluated using different measures. The neural network approach was evaluated by comparing the accuracy of different neural network algorithms using the *ACC* measure which is calculated using Eq. 8. It has been shown in [62] that the best average value of *ACC* is 0.9532. The HFSTM model was evaluated by comparing it with the Google Flu Trend (GFT). It has been shown in study [48] that the HFSTM model outperforms the GFT even with no optimization. The evaluation of the prediction market was conducted using Mean Square Error (MSE) measure. It has been shown in study [64] that the MSE was lowered dramatically when using historical context with the bigram model. The best value of MSE is 40.67. For the Historical pattern method, it has been shown in [45] that the precision for 1-day prediction is 0.8 (with mean of 0.52) and 0.6 (with mean of 0.46) for 7-days prediction. The Journal/conference backgrounds of the reviewed studies are listed in Table 3.

**Table 3:** Journal/conference backgrounds of the reviewed studies

Method category	Method name	Study reference	Journal/conference background	
Graph data mining	Graph data mining	[23]	Environment and public health	
Text mining	Historical patterns	[45]	Web intelligence	
	Co-occurrences	[44]	Infection control	
Topic models	ATAM	[46]	Health	
	ATAM+	[47]	Social media	
	HFSTM	[48]	Data mining	
Machine learning	Neural network	[61]	Cognitive information processing	
	SVM	[57]	Multiple scientific disciplines	
		[56]	Natural language processing	
		[59]	Computational linguistic	
		[58]	Biology and medicine developments	
		[60]	Living system	
		[55]	Computational biology	
	Pred. market using SVR	[64]	Social media mining	
	Naive bayes	[63]	Health care	
Math/Statistical models	SNEFT	[67]	Networking systems	
	ACF	[65]	Bioinformatics	
	Numerical-based analysis (SEHA using BOW)	[68]	Sustainable global development	
Mechanistic disease models	Metpopulation model	[70]	World wide web	
	Compartmental model	[48]	Data mining	
		[35]	Multiple scientific disciplines	
	Agent-based model	[73]	Healthcare informatics	
Keys/Documents filtration	Keys/Documents filtration	[74]	Repository of pre-prints	

## Challenges

Using social media data for disease outbreak detections call for certain challenges to be addressed [76–80].

### Data collection

The first challenge is the restriction on data collection. Social media providers use unknown and undocumented sampling filtration algorithms that allow for collecting only a sample of the overall data. In addition, there are restrictions on some private data that may be needed for the detection process. Also, users may not include some other important information. This may lead to inaccurate results produced by the tools of disease trend detection.

### Data size

The size of social media data is another issue. Today, social networking sites have become very popular and have millions of users. This would make it difficult to process such size of data by certain techniques.

### Language

The used language in social networking sites is usually informal and sometimes with spelling mistakes. Users may spell one word in different ways.

### Heterogeneity

Social media is heterogeneous. It has different kinds of users with different capabilities, activities, ages, and languages. This leads to the need for awareness of what to analyze using the data of social networking sites.

### Sampling bias

One of the serious challenges is the bias of data samples. The user population of social networking sites may not represent a sample of a society [78–80]. Alan Mislove et al. [78] analyzed the data of a very large number of Twitter users from United States to compare the Twitter population to the actual one. It has been shown in the study that the twitter users are not a random sample of the whole population and misrepresent the real distribution of race or ethnicity. Understanding this challenge will help in correcting the prediction process using social networking data if there is any bias. The correction process includes using different methods of bias quantification for further analysis and adjustment [79].

### Dataset consistency

Social media providers such as Twitter don’t allow sharing collected datasets. This is a limitation when it comes to comparing between a new proposed method and the existing ones. It is required to use consistent datasets for fair comparisons.

### User location

There is a lack of accurate user locations in SNS. A user may not share location information. In addition, the users who release this information may not update it when moving or visiting a different place.

### Proxy population

There are difficulties of defining a target population for the purpose of analysis. Populations are not self-labeled. Therefore, researchers tend to use proxy populations such as all users who use pain relievers to study the impact of pain. It has been shown that using proxy population is biased and may lead to incorrect results [79].

### Spams

There are many spam accounts that appear as normal and are frequently used to post about different topics. Researchers should be aware about these accounts and find a way to exclude them when analyzing SNS data.

### Evaluation

Evaluation is a challenging process. CDC ILINet data can be used as a ground truth for the Influenza trend detections but there is lack of ground truth for some other diseases.

## Conclusion

Social networking sites have become part of people’s lives. This has provided researchers with the opportunity to conduct different studies and researches to enhance event detections and prediction process from the data of social networking sites. In the public health area, the data of social networking sites can be used to provide early warnings of disease outbreaks such as seasonal influenza. The survey shows that the researchers have developed various methods and frameworks of flu trend detection from social networking sites. From the survey, we conclude that the research in this area is still active. More methods and frameworks may be developed to improve the accuracy of the results which can potentially be used for other disease outbreak detections for better public health.

## Abbreviations

ACF: Autocorrelation function
ARMA: Auto regression moving average
ATAM: Ailment topic aspect model
CDC: Center of disease control and prevention
GFT: Google flu trend
HFSTM: Hidden flu-state from tweet model
HPA: Health protection agency
ILI: Influenza like illness
PAHO: Pan American Health Organization
SNS: Social networking sites
SNEFT: Social network enabled flu trends
